# Early results on the use of biomaterials as adjuvant to abdominal wall closure following cytoreduction and hyperthermic intraperitoneal chemotherapy

**DOI:** 10.1186/1477-7819-8-72

**Published:** 2010-08-20

**Authors:** Cherif Boutros, Ponandai Somasundar, N Joseph Espat

**Affiliations:** 1 Hepatobiliary and Surgical Oncology, Roger Williams Medical Center, Providence, RI, USA

## Abstract

**Background:**

Hyperthermic chemotherapy applies thermal energy to both abdominal wall as well as the intra-abdominal viscera. The combination of the hyperthemia, chemotherapy and cytoreductive surgery (CRS) is associated with a defined risk of abdominal wall and intestinal morbidity reported to be as high as 15%, respectively to date, no studies have evaluated the use of biomaterial mesh as adjuvant to abdominal wall closure in this group of patients. In the present report, we hypothesized that post HIPEC closure with a biomaterial can reduce abdominal wall morbidity after CRS and hyperthermic intraperitoneal chemotherapy.

**Materials and methods:**

All patients treated with HIPEC in a tertiary care center over 12 months (2008-2009) period were included. Eight patients received cytoreductive surgery followed by HIPEC for 90 minutes using Mitomycin C (15 mg q 45 minutes × 2). Abdominal wall closure was performed using Surgisis (Cook Biotech.) mesh in an underlay position with 3 cm fascial overlap-closure. Operative time, hospital length of stay (LOS) as well as postoperative outcome with special attention to abdominal wall and bowel morbidity were assessed.

**Results:**

Eight patients, mean age 59.7 ys (36-80) were treated according to the above protocol. The primary pathology was appendiceal mucinous adenocarcinoma (n = 3) colorectal cancer (n = 3), and ovarian cancer (n = 2). Four patients (50%) presented initially with abdominal wall morbidity including incisional ventral hernia (n = 3) and excessive abdominal wall metastatic implants (n = 1). The mean peritoneal cancer index (PCI) was 8.75. Twenty eight CRS were performed (3.5 CRS/patient). The mean operating time was 6 hours. Seven patients had no abdominal wall or bowel morbidity, the mean LOS for these patients was 8 days. During the follow up period (mean 6.3 months), one patient required exploratory laparotomy 2 weeks after surgery and subsequently developed an incisional hernia and enterocutaneous fistula.

**Conclusion:**

The use of biomaterial mesh in concert with HIPEC enables the repair of concomitant abdominal wall hernia and facilitates abdominal wall closure following the liberal resection of abdominal wall tumors. Biomaterial mesh prevents evisceration on repeat laparotomy and resists infection in immunocompromised patients even when associated with bowel resection.

## Introduction

Hyperthermic intraperitoneal chemotherapy (HIPEC) has emerged as an effective method of managing peritoneal carcinomatosis for different abdominal malignancies particularly of colorectal and ovarian origin [1-3]. Most HIPEC treated patients have had prior abdominal surgeries and a substantial group of them present with abdominal wall morbidities including incisional hernia prior to HIPEC therapy.

**Figure 1:**
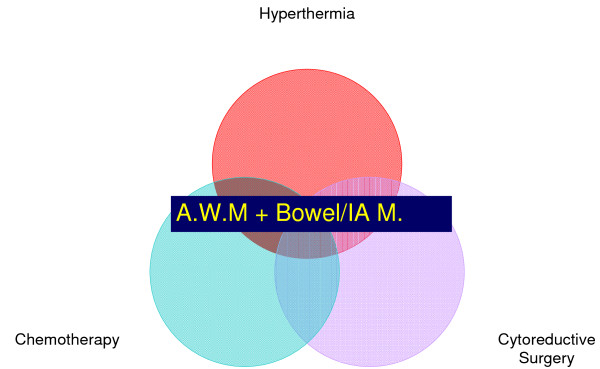
**The association of hyperthermia, cytoreductive surgery and chemotherapy carry a considerable risk of abdmonial wall and bowel morbidity**.

Multiple studies have reported that chemotherapy administration impairs wound healing and that there is associated increase in wound complications in chemotherapy exposed patients [4,5]. The effects of HIPEC on wound healing are multiple (Figure 1): first HIPEC requires the concomitant use of chemotherapy and hyperthermia which are both known to increase cellular death and induce apoptosis [6,7]; second, tumor cytoreduction may require surgical resection of involved abdominal wall surfaces potentially compromising abdominal wall closure and strength.

HIPEC impairment of wound healing is not limited to the abdominal wall, but also anastomosed segments of the gastrointestinal tract after cytoreductive surgery bowel resections. In small animal models, HIPEC was found to significantly decrease both colonic anastomotic bursting pressure and abdominal wall strength in association with decreased local protein production [8,9]. In clinical studies, HIPEC was associated with a non negligible percentage (up to 15%) of abdominal wall morbidity including; wound infection, dehiscence, evisceration and bowel morbidity including; anastomotic leak and intra-abdominal abscess (Table 1) [10,11].

**Table 1:** Selected studies reporting abdominal wall morbidity (AWM) and bowel/intra-abdominal morbidity (Bowel/IA M)

Study (year)	N	AWM	Bowel/IA M
Franko (2008) [10]	65	10.7%	15.4%
Kianmanesh (2007) [2]	43	11.6%	13.9%
Stewart (2006) [11]	110	15.4%	6.3%
Sugarbaker (2006) [29]	356	3%	5.47%
Witkamp (2001) [30]	29	3%	3%

Moreover, postoperative bowel morbidity often required reoperation [10], in this case abdominal wound healing already inhibited by HIPEC will be impaired by the infected milieu.

Biomaterial mesh has emerged as an attractive option for complex abdominal wall reconstruction providing an additional reinforcement to the abdominal wall with an absorbable material resistant to infection and with potential remodeling to host own tissue. Therefore, we used a biomaterial mesh to reinforce the abdominal wall closure at the end of CRS-HIPEC procedure. We hypothesized that this approach could minimize the rate of postoperative abdominal wall complications reported after CRS-HIPEC.

## Methods

Under institutional IRB approval, all patients treated with HIPEC at a tertiary care center over a12 months interval (2008-2009) period were identified using a prospectively maintained departmental database. Records were reviewed for preoperative, operative and postoperative data. Pertinent information for analysis included gender, age, primary malignancy, number of previous abdominal operations, peritoneal cancer index [12], operative time and cytoreductive procedures performed. Follow-up data were obtained by review of clinic notes. Abdominal wall and bowel/intra-abdominal morbidities were ascertained during the immediate and postoperative period and subsequent clinic visits.

Data was recorded in a Microsoft Excel^® ^(Microsoft, Redmond, WA) database. Descriptive statistics, including means, standard deviations or counts and percentages were calculated.

### Operative technique

Abdominal exploration was performed through midline laparotomy incision. Lysis of adhesions, when needed was performed, followed by assessment of the intra-abdominal extent of the disease and the peritoneal cancer index (PCI). Appropriate cytoreductive surgery was then performed with an attempt for complete removal of all macroscopic tumor deposits on parietal and visceral peritoneal surfaces and resection of involved viscera.

**Figure 2:**
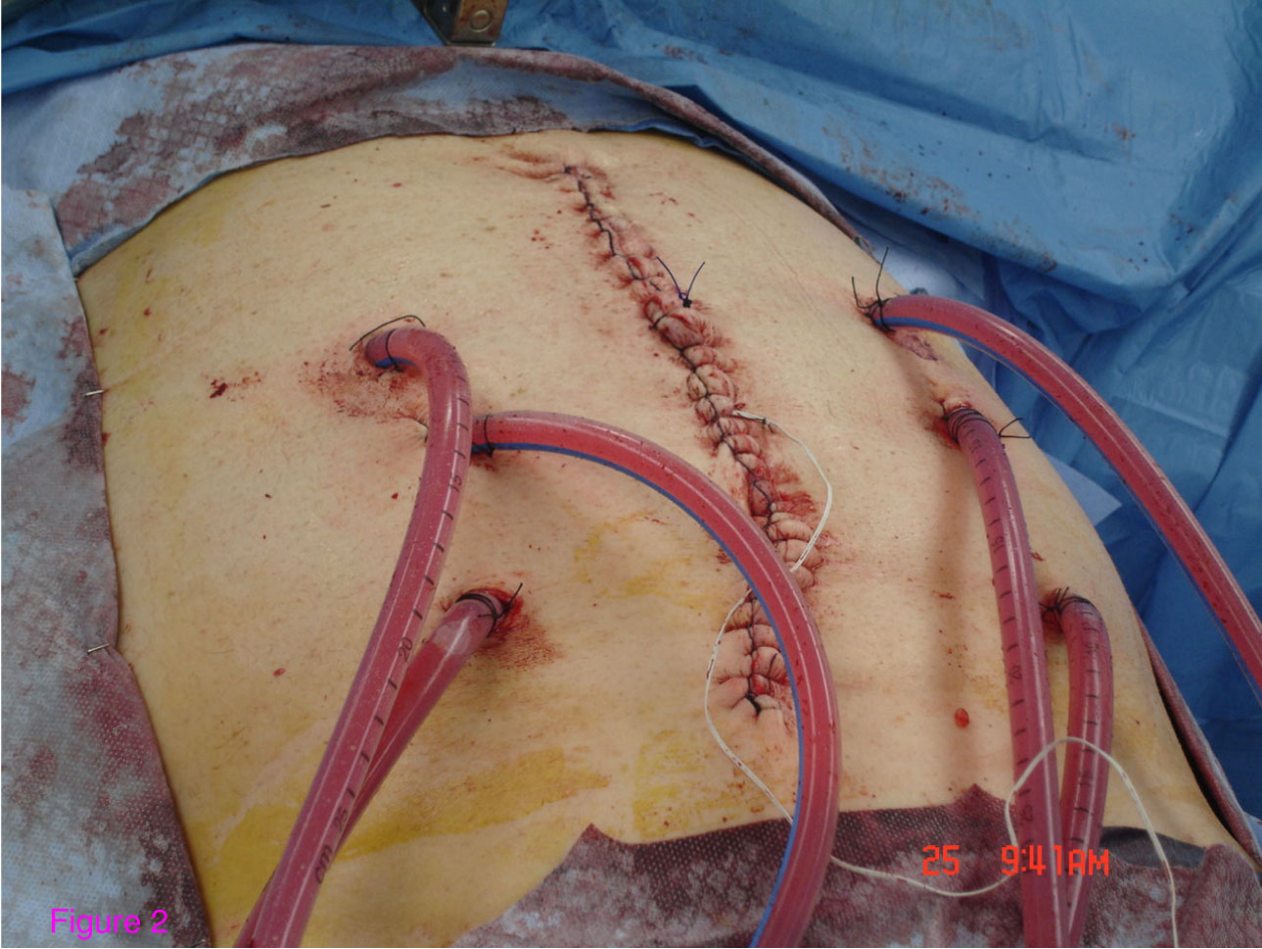
**Using a closed technique: Two inflow and four outflow catheters were used for HIPEC administration. Only the skin of the surgical wound is temporarily closed**.

**Figure 3:**
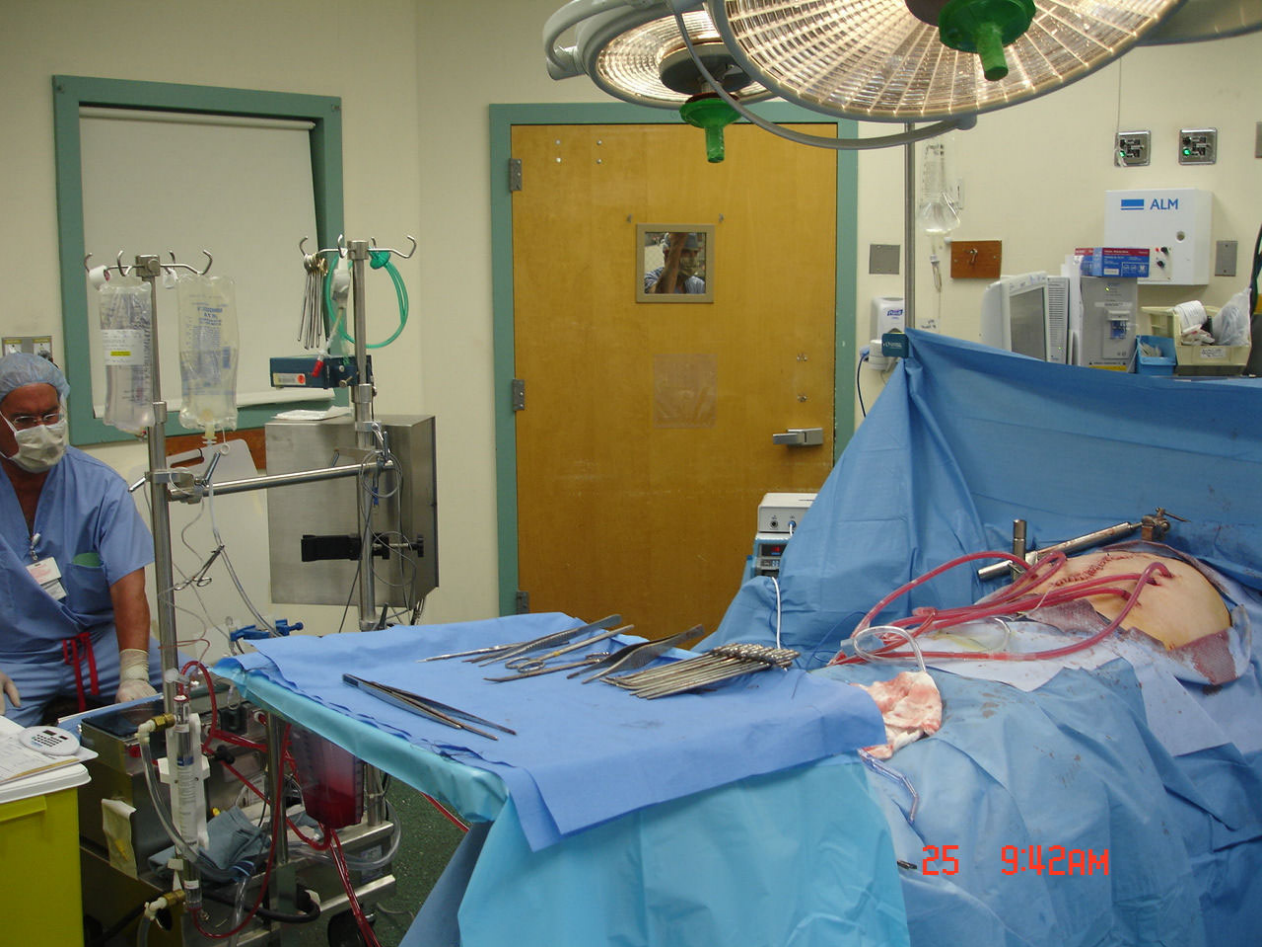
**Using a cardiopulmonary bypass machine, The HIPEC circuit is completed**.

After completion of the CRS, HIPEC was immediately performed during the same surgical procedure for all patients using a closed-abdomen technique (Figure 2). Two inflow catheters were placed under direct vision to the right and left upper quadrants above the liver and the spleen respectively and two outflow catheters were placed under direct vision on the right and left para-colic gutters. The inflow and outflow tubes were secured to the skin and connected to a cardiopulmonary bypass pump (Figure 3). A temperature needle probe was placed into one of the outflow catheters and the skin was closed using a running locked heavy nylon stitch, while the fascia was left open.

After priming the circuit, a flow of 2.0-2.4 L/minute of DIANEAL PD-2, dextrose 1.5%^® ^(Baxter, Deerfield, IL) solution containing Mitomycin C was obtained and maintained during the whole HIPEC time. Mitomycin C was used at a standard dose of 15 mg q 45 minutes × 2 for all patients. Hyperthermia was obtained by a heating unit in the cardiopulmonary bypass pump maintaining inflow temperature at 42°C, while the abdominal temperature was continuously checked by the needle probe to confirm a minimu of 41°C in the outflow. A total HIPEC time of 90 minutes was applied to all patients.

At the end of the 90 minutes dwell time, the HIPEC solution was retrieved through the cardiopulmonary pump outflow catheters and disposed; the abdominal cavity was flushed with the effluent removed via the outflow catheters and the abdominal incision was reopened.

The abdominal wall was closed using a 20 × 20 cm piece of Surgisis^® ^mesh (Cook Biotech, West Lafayette, IN). Abdominal wall closure was performed by the same surgeon performing the CRS-HIPEC procedure. Surgisis mesh was placed in underlay position and secured to the anterior abdominal wall using circumferential transfascial absorbable sutures (#1 PDS) 2 cm apart. When possible, the native fascia was closed over the Surgisis mesh using absorbable sutures and the skin was closed using skin stapler.

## Results

The RWMC HIPEC program started on June 2008; over one year period eleven patients received HIPEC, including three patients who received totally laparoscopic HIPEC for persistent PET scan evident activity from colorectal cancer in the mesenteric or retroperitoneal lymph nodes after standard adjuvant chemotherapy. Eight patients received exploratory laparotomy, CRS and HIPEC and were included in this study (Table 2).

**Table 2:** Patient characteristics

Age	Sex	ASA	Pathology	Prior surgery	PCI
53	M	2	MAC Appendix	Appendectomy	18
70	M	2	Colorectal cancer	Open colectomy and ventral hernia repair	3
59	F	3	Ovarian Cancer	Three midline Ex Lap.	12
57	F	2	MAC Appendix	Appendectomy	0
72	M	3	Colorectal Cancer	Open colectomy	9
80	F	3	Ovarian Cancer	Hysterectomy	10
36	F	2	MAC Appendix	TAHBSO	15
51	M	3	Colorectal cancer	Open colectomy + 4 Ex. Lap.	3

PCI: Peritoneal cancer index; MAC: Mucinous adenocarcinoma; TAHBSO: total abdominal hysterectomy and bilateral salpingo-oopherectomy; Ex. Lap.: Exploratory laparotomy.

Patients' mean age was 59.7 years (36-80), M: F ratio was 1:1 and the origin of the primary malignant disease was colorectal (n = 4), appendiceal (n = 2) and ovarian (n = 2). All patients had prior abdominal surgeries and three patients had > two prior abdominal explorations. Peritoneal cancer index varied from 0-18 with a mean of 8.75. Prior to HIPEC therapy, four patients (4/8, 50%) presented with abdominal wall morbidities including incisional hernias (n = 3) and abdominal wall metastatic implants (n = 1).

A total 28 CRS procedures were performed (average 3.5/patient). One third of the CRS included bowel resection-anastomosis (9/28, 32%) all performed by linear staplers.

Mean total surgical procedure time was 5 h 49 minutes ± 1 h 10 minutes including 90 minutes devoted for HIPEC (Table 3). There was no peri-operative mortality and no postoperative neutropenia. Seven patients had neither abdominal wall nor bowel/intra-abdominal morbidities with a mean length of stay of 8 days (range 4-16). All patients were followed in subsequent clinic visits, the mean follow up was 6.3 months.

**Table 3:** Outcome of patients after HIPEC surgery

Surgical time (mn)	AWM	Bowel/IA M	LOS (D)
410	ø	ø	9
310	ø	ø	4
380	Dehiscence	ECF	120
280	ø	ø	6
370	ø	ø	5
430	ø	ø	16
230	ø	ø	10
380	ø	ø	6

AWM: Abdominal wall morbidity; Bowel/IA M: Bowel and intra-abdominal morbidity; LOS: Length of stay; ECF: Enterocutaneous fistula

One patient required re-exploration two weeks after HIPEC procedure and subsequently developed incisional hernia and enterocutaneous fistula. This patient is a 59 year old patient with ovarian cancer and three prior abdominal surgeries including two debulking procedures. Prior to HIPEC procedure, the patient physical examination was significant for extensive abdominal wall metastatic implants.

The patient PCI was 12 and CRS included bowel resection/anastomosis x2, splenectomy and abdominal wall resection. The patient post-operative period was marked by respiratory failure requiring reintubation. On post-operative day 15, a brown discharge was noted from the surgical incision and a decision for surgical re-exploration was made.

Upon re-exploration, the previously placed Surgisis mesh was intact and easily dissected from the underlying bowel, prior anastomoses were intact and there was no evidence of gastrointestinal leak. Blood culture was obtained when the postoperative wound discharge was initially noted and intravenous antibiotic therapy was started imperatively with the hypothesis of sepsis. During re-exploration cultures from the peritoneal fluid were also obtained; both blood and peritoneal cultures did not support an infectious etiology. A dramatic lysis of residual tumor implants was noted producing a melted brown discharge. A new Surgisis mesh was placed and the fascia was closed as previously described. Subsequently, the patient developed wound dehiscence with no evisceration as well as enterocutaneous fistula. A negative pressure dressing was applied to the abdominal wound and the patient was discharged home three months after the HIPEC procedure.

## Discussion

The introduction of biomaterial mesh had revolutionized the field of abdominal wall closure [13,14]. Broadly grouped, biomaterial mesh is either human allograft or xenograft and dermal or non dermal in origin. Specific guidelines for specific biomaterial mesh selection for a given case remain to be defined; however in general it is accepted that for complex and contaminated cases, biomaterial mesh offers a viable substitute to the patient's own tissue. In particular the use of biomaterial mesh has been described to be clinically meaningful when the host native abdominal fascia is insufficient for closure without tension, ie (loss of abdominal domain), when there is a lack of viable tissue and a components separation is not technically feasible, or the field is contaminated or potentially contaminated and permanent synthetic mesh is relatively contraindicated [15]. Biomaterial meshes are known to be resistant to infection[16] and overcome the limitations of synthetic mesh for use in contaminated or potentially contaminated wounds, provide a tissue remodeling matrix, for host tissues and fibroblasts [17]. In this series, the potential role of biomaterial mesh as adjuvant to abdominal wall closure in the setting of significantly potential impaired abdominal wall wound healing following HIPEC, with or without prior incisional hernia or after cytoreductive surgery of abdominal wall metastatic implants was investigated. In cases, there was a clinical indication for mesh reinforcement due to weakened, lacking or non viable abdominal wall fascia; the choice of biomaterial mesh was supported by the presence of potential contamination or frank contamination subsequent to a procedure entering the gastrointestinal tract.

Surgisis mesh was utilized in all open HIPEC procedures. This biomaterial mesh is composed of lyophilized porcine small intestinal submucosa, is known to attract cells to the wound area and signaling surrounding tissues to grow across the scaffold [18]. The choice of this particular biomaterial mesh was based on the senior authors previous published experience with Surgisis [14,19]as well as the reports of others observing that Sugisis remodels into vascularized host tissue [17], thus allowing resistance to infection. Additionally, Surgisis is predominantley composed of collagen rather than elastin compared to dermal-based biomaterials; thus it is expected to result in less abdominal diathesis or hernia recurrence overtime [20].

All Surgisis meshes were placed in underlay position with a minimum of a bilateral 3 cm fascial overlap-closure using absorbable number one PDS transfascial sutures placed circumferentially no more than two cm apart. Underlay repairs, such as Rivers-Stoppa retro-rectus repair, have been reported to result in improved recurrence rate and allow for re-approximation of the midline, thus potentially improving the mechanical function of the abdominal wall [21,22].

The HIPEC protocol employed was the well-described regimen of single agent (Mitomycin C) at a dose (15 mg q 45 minutes x2) with a cumulative dwell time of 90 minutes, for all patients regardless of the origin of the primary malignancy and the body surface area of the patient [23]. The present protocol does not include a measurement of serum Mitomycin C levels thus we are unable to discuss its pharmakodynamics in this setting, these data have been previously described by others [24,25]. Postoperatively, none of the patients in this series developed neutropenia which has reported to occur in up to 39% of HIPEC patients using Mitomycin C at a higher dose[26]. It should be emphasized that though there are multiple series reporting the use of different chemotherapeutic agent (s) and different doses during HIPEC, there is no consensus statement or general agreement on a single universal protocol. Currently, efforts are undergoing to create a registry database for all active HIPEC programs in USA allowing outcome analysis to elucidate this still evolving topic.

Seven of eight patients included in this study did not develop abdominal wall or bowel/intra-abdominal morbidities postoperatively and were discharged home after a mean length of stay of eight days. A single patient did sustain the complication of suspicious for enterocutaneous fistula wound discharge with associated with respiratory failure 7 days after HIPEC necessitating re-exploration. Upon re-exploration, the integrity of the abdominal wall and gastrointestinal were verified. The Surgisis mesh was found intact and the bowel was easily dissected from the mesh as has been previously described in experimental models [27,28]. At operation, the patient was found to have extensive tumor necrosis from unresectable pelvic mass; because of potential compromised rectal wall, a loop diverting ostomy was created. The abdominal wall fascia was closed again with a new Surgisis mesh to prevent evisceration. Not unexpectedly, the native fascia later dehisced, and the patient developed and enterocutaneous fistula, however, the new Surgisis mesh was allowed to granulate and subsequently remodeled, allowing non surgical management.

Subsequent to HIPEC, perioperative abdominal wall and bowel/intra-abdominal complications may require surgical exploration [10]. In this population re-exploration carries a high risk to both the abdominal wall recently exposed to hyperthermic chemotherapy and the bowel is usually firmly adherent to the abdominal with associated evisceration and/or the formation of enterocutaneous fistulae. The use of synthetic mesh as adjuvant to abdominal wall closure in such circumstances is contraindicated due to the potentially infected and contaminated environment.

The authors would emphasize the limitations of this study. This series represents early results of an abdominal wall reconstructive technique that in our experience was found to be safe and effective in preventing abdominal wall complications in a cohort of patients at high risk in the short term postoperative period. However, this report represents the experience of a single institution with a modest cohort and a short follow up. Long term follow up is needed before complete success for the use of biomaterial mesh can be declared to more define the role for biomaterial mesh in treatment and prevention of surgical site hernia following HIPEC and cytoreductive surgery. This may be limited by the inherent limited survival of many patients with disseminated peritoneal malignancy.

In this small series biomaterial mesh was applied at the end of all CRS-HIPEC procedure and showed a beneficial effect in minimizing early postoperative abdominal wall complications. However, extrapolating these results to be applied to all CRS-HIPEC patients as a universal approach will probably reveal that only a group of high risk patients including those with multiple abdominal wall surgeries, preoperative abdominal wall hernia or metastatic implants to the abdominal wall will benefit from such approach.

The selection of Surgisis mesh, was based on results of other studies reporting its resistant to infection and total remodeling in settings other than HIPEC. It will be necessary to document remodeling in this setting by histopathology incorporating tissue samples during the various stages of wound healing at distinct intervals after HIPEC with and without Surgisis mesh abdominal wall reconstruction.

Finally, the HIPEC protocol used only Mitomycin C as single agent chemotherapy. Although, chemotherapeutic agents share an inhibitory effect on wound healing, it is possible, although not yet investigated that in the setting of HIPEC, that this known inhibitory effect varies significantly between different agents.

## Conclusion

In this series, the use of a biomaterial mesh for abdominal wall reconstruction following CRS and HIPEC enabled the repair of concomitant abdominal wall hernias and facilitated liberal resection of abdominal wall tumors. Biomaterial mesh prevented evisceration on repeat laparotomy and resists infection in immunocompromised patients even when associated with bowel resection.

## Competing interests

The senior author, Dr. Espat has previously received honoraria from Cook as a speaker.

## Authors' contributions

CB assisted with acquisition of data and drafting the manuscript, PS assisted with revision of the manuscript, NJE assisted with study design and revision of the manuscript. All authors read and approved the final manuscript
